# Artificial Ageing, Chemical Resistance, and Biodegradation of Biocomposites from Poly(Butylene Succinate) and Wheat Bran

**DOI:** 10.3390/ma14247580

**Published:** 2021-12-09

**Authors:** Emil Sasimowski, Łukasz Majewski, Marta Grochowicz

**Affiliations:** 1 Department of Technology and Polymer Processing, Faculty of Mechanical Engineering, Lublin University of Technology, Nadbystrzycka 36, 20-618 Lublin, Poland; e.sasimowski@pollub.pl; 2 Department of Polymer Chemistry, Institute of Chemical Sciences, Faculty of Chemistry, Maria Curie-Sklodowska University, M. Curie-Sklodowska 3, 20-031 Lublin, Poland; mgrochowicz@umcs.pl

**Keywords:** accelerated ageing, biofiller, thermal properties, agro-waste materials, agro-flour filler, natural filler, thermal resistance, discolouration, lignocellulosic materials, biopolymer, composites

## Abstract

The results of comprehensive studies on accelerated (artificial) ageing and biodegradation of polymer biocomposites on PBS matrix filled with raw wheat bran (WB) are presented in this paper. These polymer biocomposites are intended for the manufacture of goods, in particular disposable packaging and disposable utensils, which decompose naturally under the influence of biological agents. The effects of wheat bran content within the range of 10–50 wt.% and extruder screw speed of 50–200 min^−1^ during the production of biocomposite pellets on the resistance of the products to physical, chemical, and biological factors were evaluated. The research included the determination of the effect of artificial ageing on the changes of structural and thermal properties by infrared spectra (FTIR), differential scanning calorimetry (DSC), and thermogravimetric analysis (TG). They showed structural changes—disruption of chains within the ester bond, which occurred in the composition with 50% bran content as early as after 250 h of accelerated ageing. An increase in the degree of crystallinity with ageing was also found to be as high as 48% in the composition with 10% bran content. The temperature taken at the beginning of weight loss of the compositions studied was also lowered, even by 30 °C at the highest bran content. The changes of mechanical properties of biocomposite samples were also investigated. These include: hardness, surface roughness, transverse shrinkage, weight loss, and optical properties: colour and gloss. The ageing hardness of the biocomposite increased by up to 12%, and the surface roughness (R_a_) increased by as much as 2.4 µm at the highest bran content. It was also found that ageing causes significant colour changes of the biocomposition (ΔE = 7.8 already at 10% bran content), and that the ageing-induced weight loss of the biocomposition of 0.31–0.59% is lower than that of the samples produced from PBS alone (1.06%). On the other hand, the transverse shrinkage of moldings as a result of ageing turned out to be relatively small, at 0.05%–0.35%. The chemical resistance of biocomposites to NaOH and HCl as well as absorption of polar and non-polar liquids (oil and water) were also determined. Biodegradation studies were carried out under controlled conditions in compost and weight loss of the tested compositions was determined. The weight of samples made from PBS alone after 70 days of composting decreased only by 4.5%, while the biocomposition with 10% bran content decreased by 15.1%, and with 50% bran, by as much as 68.3%. The measurements carried out showed a significant influence of the content of the applied lignocellulosic fillers (LCF) in the form of raw wheat bran (WB) on the examined properties of the biocompositions and the course of their artificial ageing and biodegradation. Within the range under study, the screw speed of the extruder during the production of biocomposite pellets did not show any significant influence on most of the studied properties of the injection mouldings produced from it.

## 1. Introduction

Plastic is one of the most popular construction materials today. Its annual global production is counted in hundreds of million tons [1,2,3,4]. The scale of plastic use results in significant environmental pollution, which has been a large problem for many years [3,5,6,7]. Nevertheless, it is predicted that global plastic production may even double in the next 15–20 years, which may lead to inefficiencies in plastic waste disposal and recycling mechanisms [8,9,10]. Potential opportunities to reduce the scale of the problem are seen, inter alia, in the assumptions of a circular economy, mechanisms for reduction of plastic consumption, and in industrial scale popularization of biopolymers and biocomposites based on biopolymer matrix [8,11,12,13,14,15,16,17].

Biopolymers are mainly materials capable of biodegradation in environmental conditions with participation of microorganisms, as this term is also used for non-biodegradable polymers obtained from substrates of natural origin [18,19,20]. The kinetics of polymer degradation depends mainly on the chemical structure of macromolecules, which translates into their thermal resistance, chemical activity, or resistance to oxidation or action of acids, bases, and enzymes. The chemical structure of a polymer will therefore directly affect its susceptibility to degradation caused by various external factors [21,22,23,24,25,26]. Depending on the sensitivity of the material to the particular stimulating factors, other types of degradation are distinguished in addition to microbial biodegradation. These include thermal degradation at elevated temperatures, mechanical degradation caused by long-term stress, oxidative degradation in oxygen-containing atmospheres, photodegradation caused by light rays, hydrolytic degradation at high humidity, corrosion caused by the activity of chemical substances, and the effect of high-energy electromagnetic radiation (e.g., UV) [20,27,28,29,30]. The above-mentioned physical and chemical factors lead to irreversible changes in the material structure, such as a change in the proportion of the crystalline phase or decomposition of macromolecules into shorter chains, and consequently, also to changes in mechanical and physical properties [31,32,33,34]. These changes can be manifested, e.g., by an increase in the stiffness and brittleness leading to the fragmentation of material and an increase in the surface area affected by external factors, but also by a loss of mass or a change in the colour and roughness of the surface [35,36,37,38,39]. Therefore, the activity of physical and chemical agents significantly accelerates the degradation process of biopolymers increasing the specific surface area of their influence by fragmentation of the material. However, the actual process of degradation and mineralization of biopolymers to simple substances such as water, carbon dioxide, and inorganic compounds takes place primarily through the activity of microorganisms [28,29,37,40,41,42]. Different biopolymers have different susceptibility to degradation agents and different degradation rates [43]. Water-soluble biopolymers, such as poly(vinyl alcohol) or starch-based plastics, will degrade most rapidly in an aqueous environment [44,45,46]. Some polymers, with adequate moisture content, biodegrade relatively quickly at room temperature or slightly higher by incubation with mesophilic bacteria. The latter include polyhydroxyalkanoate and polyhydroxybutyrate [47,48]. There is also a group of biopolymers that require appropriate conditions for proper and quick biodegradation process. Such factors may include the appropriate level of humidity, the presence of specific strains of bacteria and the pH that is adequate for them, or an elevated temperature corresponding to thermophilic microorganisms. Examples of such biopolymers include polycaprolactone, polylactide, and poly(butylene succinate) (PBS) [37,49,50,51,52,53]. Resistance to external factors has a direct impact on the choice of biopolymer for specific applications and the life cycle of polymer products [54,55,56].

The life cycle encompasses all stages related to the presence of a given product on the market, from designing, manufacturing, exploitation, and management of waste generated during its use. The duration of each stage varies depending on the type of product and the material used [54,55,56,57,58]. In the case of polymeric materials of petrochemical origin, the landfilling stage of waste management, the process of natural decomposition of polyolefins, for example, takes up to several hundred years. This is a direct cause of the ever increasing environmental pollution from plastic waste [59,60,61]. The use of biodegradable polymers, especially those that degrade under conditions other than their operating conditions, allows the situation to be reversed. Under standard conditions, Poly(butylene succinate) can be exploited for a long time, and when directed to composting under conditions stimulating biodegradation, it decomposes within a few months [52,62]. This in combination with mechanical properties comparable to polyolefins as well as good processability and thermal resistance make PBS an attractive alternative for polymers of petrochemical origin [63,64,65]. However, PBS has a multi-stage and relatively complex manufacturing process, which at the same time is very expensive, drastically increasing the price of PBS as a raw material [64,66,67,68,69]. In addition, running such complex production processes on an industrial scale cannot be done without an environmental footprint [70,71]. Therefore, despite the biodegradability of PBS, it is justifiable to apply mechanisms to minimize the consumption of raw materials and energy during manufacturing, both ecologically and economically. This is done, among others, by filling PBS with lignocellulosic fillers (LCF) of natural origin, which are most often technological wastes from food and agricultural industry. This reduces the consumption of expensive plastic while using waste from other economic sectors [72,73]. Examples of such waste fillers used to produce PBS matrix biocomposites include ground rice husks [74], wheat bran [75], pistachio [76] and peanut shells [77], almond kernels [78], or even wine lees [79], as well as apple [80], and grape pomace [81]. The presence of LCF significantly modifies the physical, mechanical, thermal, and processing properties of such compositions [72,82,83,84]. In addition, due to their chemical structure, LCFs display hydrophilic character and are much less resistant to physical and chemical factors and microbial activity compared to PBS or other polymeric materials [85,86]. This may be a limiting factor, resulting in a shorter life cycle for products made from PBS/LCF biocomposites and a reduced spectrum of potential applications, but also imply simplified disposal through accelerated biodegradation. Therefore, when designing new composite systems and using new types of fillers, it is important to know the full characteristics of the material, taking into account processability, physical, mechanical and thermal properties, chemical resistance and resistance to ageing, as well as kinetics and course of biodegradation.

In spite of numerous papers dealing with biodegradable polymeric materials, the current literature lacks studies on the characterization of PBS-based biocomposites with a filler in the form of raw wheat bran (WB). A biocomposite with such a composition undergoing natural decomposition is the subject of a patent [87] and constitutes a completely new material. Consequently, the results of testing the resistance of PBS/wheat bran biocomposites to external factors presented in this paper are a scientific novelty and, at the same time, will be helpful in determining the spectrum of applications of the new biocomposite. The aim of this work was to evaluate the influence of wheat bran content in the range 10–50 wt.% and extruder screw speed in the range 50–200 min^−1^ during the production of composite pellets on the resistance of the manufactured biocomposite injection mouldings to chemical, biological, and physical factors. Changes of selected structural, thermal, mechanical, and optical properties of biocomposites, which occurred as a result of artificial ageing, were also evaluated.

## 2. Experimental Procedures

### 2.1. Test Stand

Injection moulding of the biocomposite was carried out using an Arburg Allrounder 320 C (Arburg, Lossburg, Germany) screw injection moulding machine equipped with a dual cavity mould to produce specimens for strength testing. The shape and dimensions of the samples were in accordance with ISO 294-1:2017-07 [88]. The specimens were dog-bone-shaped with a total length of 150 mm and a thickness of 4 mm; the width of the measuring part was 10 mm, and the grip part was 20 mm. Due to the danger of thermal decomposition of biocomposite components, low temperatures were applied during processing. The temperature of the plasticizing system was 30 °C in the feed zone, and in the individual heating zones: I—125 °C, II—145 °C, III—155 °C, IV—160 °C, and the injection nozzle temperature was 155 °C. The temperature of the thermostated mould was 25 °C. The injection of the biocomposition was performed at the following settings: maximum injection pressure 120 MPa, polymer flow rate 20 cm^3^/s, packing pressure 110–80 MPa, packing time 15 s, and cooling time 20 s. In the case of the highest bran fraction of 50% (DOE layout 8), the injection pressure was increased to 130 MPa and the packing pressure to 120–80 MPa, which made it possible to eliminate incomplete filling of mould cavities, occurring at lower values of these parameters.

### 2.2. Materials

The prepared test samples were based on employing as matrix PBS with the trade name BioPBS FZ91 PB [89], manufactured in the form of pellets (PTT MCC BIOCHEM CO., LTD, Bangkok, Thailand). It was synthesized using bio-based succinic acid and 1,4-butanediol. This material is intended for manufacturing general-purpose products via injection moulding.

Wheat bran (WB), i.e., wheat grain shells, is a process waste product from the refining of white flour. It comes in the form of thin flakes with dimensions up to a few mm and was obtained from a local mill near the city of Lublin (Poland). WB is primarily composed of fibrous substances such as cellulose, lignin and hemicellulose, but includes phytic acid, oligosaccharides, non-starch polysaccharides, as well as fats and proteins [90,91].

### 2.3. Research Programme and Methodology

Experimental tests were carried out according to the adopted design of experiment (DOE), the experimental layouts of which are shown in Table 1. The following independent variables—adjustable conditions of the process—were assumed: mass content of wheat bran introduced into poly(butylene succinate) *u* = 10–50 wt.% and extruder screw speed *n* = 50–200 min^−1^ when obtaining processed biocomposite pellets. A detailed characterization and analysis of the twin-screw extrusion process of biocomposite pellets was presented in a previous paper [75]. Measurements were made in at least five replicates. PBS and its composites with bran containing 10% (3), 30% (5), and 50% (4) of biofiller obtained at the same screw speed and the composites containing 30% of biofiller, but obtained at *n* equal to 50 min^−1^ (1) and 200 min^−1^ (2) were selected for the tests. Such a choice of test samples allows us to consider the influence of the biofiller content and *n* parameter on the values of tested parameters.

Experimental tests of injection mouldings made of polymer compositions, prepared according to DOE, were carried out and involved:

- Performing accelerated ageing test in Xenotest Alpha+ accelerated ageing chamber (Atlas, Chicago, IL, USA). The test lasted 1000 h, the samples were irradiated with a xenon lamp emitting radiation imitating solar radiation. The irradiance of 60 W/m^2^ and the daylight filter system were used. The temperature in the chamber was kept at 38 °C, humidity 50%. During irradiation, the samples were sprayed with distilled water for 18 min every 102 min. The measurement conditions were in accordance with the standard: ISO 4892-2:2013 [92]. Before and after the test, and for selected tests also during the test, the following properties, which are the determinants of resistance to artificial ageing, were measured:
  ◦ The infrared spectra (FTIR) analysis of the samples. The accelerated ageing test was stopped for 10 min each time at 250, 500, and 750 h in order to obtain the FTIR spectra of the test samples. The used parameters of the ageing test allow us to assess the behaviour of composites in artificial weathering conditions. The FTIR of tested samples were taken using Tensor 27 spectrometer (Bruker, Germany) equipped with ATR (attenuated total reflectance) module with diamond crystal. The FTIR spectra were recorded from 600 to 4000 cm^−1^ with 32 scans per spectrum and the resolution of 4 cm^−1^. The infrared carbonyl stretching region was deconvoluted into the Gaussian curves using OPUS 7.0 software;
  ◦ Differential scanning calorimetric (DSC) studies were performed on DSC 204 F1 Phoenix (NETZSCH, Günzbung, Germany) working with the NETZSCH Proteus software, in accordance with standard ISO 11357-1:2016 [93]. Each measurement was carried in three cycles: heating from −150 °C to 140 °C with heating rate of 10 K/min (I heating), cooling from 140 °C to −150 °C with cooling rate of 10 K/min, heating from −150 °C to 140 °C with heating rate of 10 K/min (II heating). Samples with mass about 10 mg were analysed in closed pierced aluminium pans in argon atmosphere with flow rate of 25 mL/min. Parameters such as melting enthalpy (Δ*H_m_*), melting temperature (*T_m_*), crystallization temperature (*T_c_*), glass transition temperature (*T_g_*), and crystallinity degree (*X_c_*) were calculated based on the obtained thermograms. The *T_g_* value was adopted as the inflection point of the DSC curve in the area of the glass transition. The *X_c_* parameter was calculated from the equation:
    (1) $$X_{c}=\left(\frac{\Delta H}{(1-u)\times \Delta H_{100\%}}\right)\times 100\%$$
    assuming that for pure PBS, Δ*H*_100%_ = 110.3 J/g [94];
  ◦ Measurements of transverse shrinkage, determined as the percentage difference in linear dimension of the specimen before accelerated ageing and after the completed accelerated ageing cycle. Recommendations were applied from the standard ISO 294-4:2018 [95];
  ◦ Thermogravimetric analysis was performed in synthetic air with the use of STA 449 F1 Jupiter (Netzsch, Günzbung, Germany) coupled with FTIR TENSOR 27 spectrometer (Bruker, Germany). The measurement conditions were as follows: temperature range of 40–800 °C, heating rate 10 K/min gas flow 25 mL/min, and sample mass approximately 10 mg. Samples were analysed in Al_2_O_3_ opened crucibles;
  ◦ Colour measurement of samples according to ASTM E308 [96], for which X-Rite Ci4200 spectrophotometer was used. The colour is described in the CIELab system, where it is specified in L*, a*, b* space. Parameter a describes the colour from green (negative values) to red (positive values), parameter b—the colour from blue (negative values) to yellow (positive values), and parameter *L* is the luminance—brightness, representing the grey scale from black to white (value 0 corresponds to black and 100 to white). The difference between two colours—two points in the three-dimensional space L*, a*, b*—is described by the relation:
    (2) $$\Delta E=\sqrt{\Delta L^{2}+\Delta a^{2}+\Delta b^{2}}$$
    in which: Δ*L*, Δ*a*, and Δ*b* denote the difference in colour parameters between the compared samples, respectively.
  ◦ The roughness parameters of samples before and after ageing were given on the basis of results obtained from the optical profiler Contur GT (Bruker, Karlsruhe, Germany). Average roughness parameter (Ra) was calculated in accordance with ASME B46.1 [97], with the use of Vision 4.20 software. Measurements were performed at room temperature, the area of 156 µm × 117 µm was scanned in three different places for each sample. After the accelerated ageing test, the exact same sections of the surface of the samples were analysed.
  ◦ Measurement of the surface gloss of the samples using an X-Rite Ci4200 spectrophotometer (X-Rite, Grand Rapids, MI, USA), performed in accordance with ISO 2813:2001 [98] at the 60° image slit opening angle for the light source and receiver.
  ◦ Weight loss measurements, determined as the percentage difference between the initial weight of dry samples and the weight of dry samples after completion of the accelerated ageing cycle;
  ◦ Hardness test using ball indentation method. The measurement was carried out in accordance with ISO 2039-1:2004 [99] using an HPK 8411 hardness tester with a ball-shaped indenter of 5 ± 0.025 mm diameter.
  ◦ Shore D hardness test was performed as per ISO 868:2003 [100] with the use of Shore durometer model ART.13 by Affri System (Induno Olona, Italy) with a cone-shaped indenter with a rounded tip.
- Assessing the absorption of polar and non-polar fluids. The tested biocomposite samples were completely immersed in water and vegetable oil for 7 days. The percentage difference in mass and linear dimensions after immersion and before immersion in the fluid was then specified to determine the values of fluid absorption and swelling. The test procedure was in accordance with ISO 175:2010 [101];
- The chemical resistance of PBS and its biocomposites was assessed in 1 M solution of NaOH and 1 M solution of HCl at room temperature. The test consisted in placing the samples (10 mm × 10 mm × 4 mm) dried to constant weight in glass bottles containing the above-mentioned solutions. The samples were taken out from solutions at specified intervals, dried on blotting paper and then weighed. The percent change of the mass was calculated according to the equation:
  (3) $$\mathrm{mass}\;\mathrm{change}(\%)=\frac{\left(m-m_{0}\right)}{m_{0}}\times 100\%$$
  where *m* is the final mass of the sample; *m_0_*—is the initial mass of the sample.
- Laboratory biodegradation tests conducted under controlled conditions as per ISO 20200:2015 [102]. Samples were placed in separate polypropylene bioreactors filled with commercially available compost from a local waste management facility. Then the bioreactors were placed in a climate chamber with a temperature of 58 °C and air humidity of 60%. At the intervals specified in the standard, water was replenished in the bioreactors and the compost was homogenized. After fixed incubation periods of 7, 14, 21, 28, 42, 56, and 70 days, respectively, the samples were extracted, washed, dried to constant weight, and weighed. On the basis of mass measurements, its loss was determined, which is an indicator of biodegradation rate.

## 3. Results

The results of the study were statistically processed in STATISTICA 13. The ANOVA variance analysis was used to determine if there were significant differences between the compared results. Before performing the above, the required assumptions such as normality of distribution of variables and homogeneity of variance were checked. Non-parametric test was applied where the variables did not meet the condition of normality of distribution. When these analyses confirmed the presence of statistically significant differences, Tukey’s multiple comparisons test was then performed. This test, called the post-hoc test, facilitates grouping of means and separation of homogeneous groups. The significance level of *p* = 0.05 was adopted in the applied analyses. The results obtained are presented in the form of graphs, where the mean values are marked together with the standard error. For most of the properties under study, the influence of both variable parameters, i.e., bran content *u*, and extruder screw speed *n*, was presented separately on graphs.

### 3.1. Accelerated Artificial Ageing

#### 3.1.1. Chemical Structure

**Figure 1:**
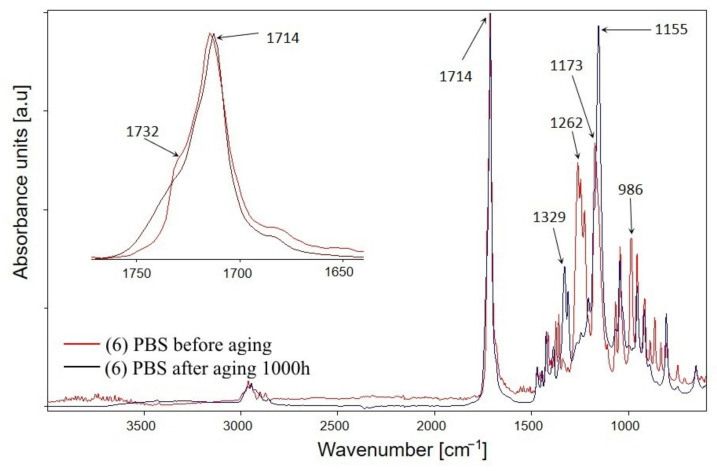
ATR-FTIR spectra of PBS acquired before and after ageing test.

**Figure 2:**
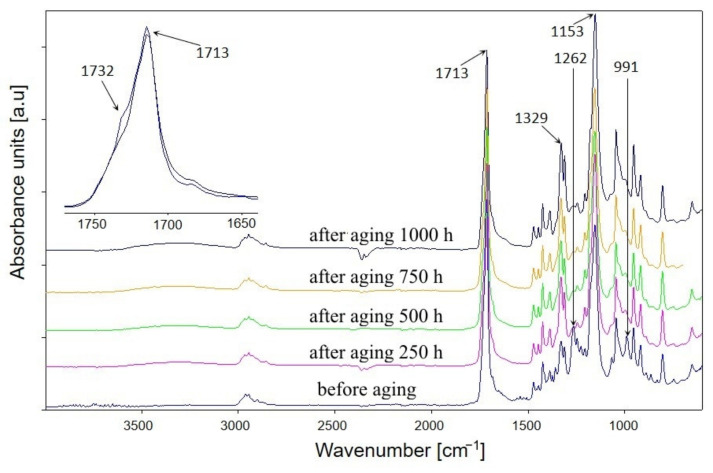
ATR-FTIR spectra of biocomposite 4 containing 50% of bran, in ageing test intervals.

**Figure 3:**
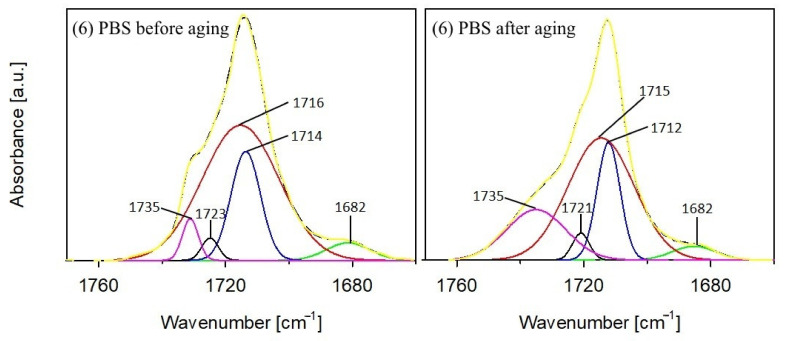
Deconvolution of the carbonyl stretching region of PBS before and after aging (dashed line: resolved peaks, solid line: recorded spectra).

**Figure 4:**
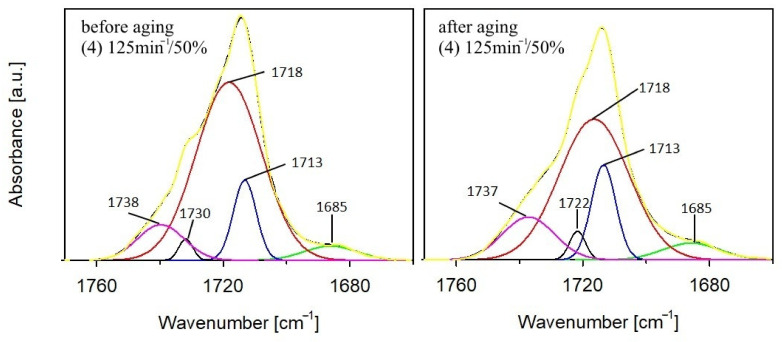
Deconvolution of the carbonyl stretching region of composite with 50% bran before and after aging (dashed line: resolved peaks, solid line: recorded spectra).

The total residence time of the samples in the ageing chamber was 1000 h, but the samples were subjected to FTIR analysis several times during the study at specific time intervals. FTIR spectra of PBS before and after ageing are presented in Figure 1, whereas FTIR spectra performed in successive hours of the ageing test for the composite containing the highest percentage of bran—sample number 4—are shown in Figure 2. The course of the spectra obtained before and after ageing is different for both PBS and composite 4. These differences are due to the structural changes that occurred in the samples as a result of artificial weathering. Figure 2 shows that the structural changes in composite 4 occurred already after the first 250 h of the composite’s stay in the ageing chamber, for the other tested materials the changes occurred in a similar way. Analysis of the FTIR spectra shows that the intensity of the absorption bands at 1262–1227 cm^−1^, which originate from the asymmetric stretching vibration of the C–O–C group, and the band at 991 cm^−1^ (in the composite) and 986 cm^−1^ (in PBS) originating from the vibration of the chain backbone have clearly decreased. Additionally, as the accelerated ageing test progresses, an increase in the intensity of the absorption bands at 1153 cm^−1^ and 1329 cm^−1^ corresponding to the symmetric stretching vibrations of the C–O–C group is observed, as well as a marked decrease in the intensity of the band at 1713 cm^−1^ originating from the vibration of the C=O group compared to the band at 1153 cm^−1^. On this basis, one may conclude that the effects of UV radiation, elevated temperature, and water result in structural changes in PBS within the ester bond. Several different mechanisms of PBS chain cleavage have been proposed in the literature; among them, α-hydrogen abstraction leading to the formation of carboxylic and aldehyde groups via hydroperoxides; the Norrish I of chain cleavage leading to carboxyl, aldehyde and ether groups; the hydroxyl end groups oxidation leading to carboxyl groups and β-hydrogen abstraction resulting in unsaturated compounds [103,104,105]. Analysis of the FTIR spectra of the composites after ageing indicates that the first three mentioned photodegradation mechanisms may have taken place. However, the lack of absorption bands from C=C unsaturated groups in the spectra excludes the possibility of β-hydrogen chain scission reaction. Moreover, considering the region of the spectra of PBS and composite 4 in the vibrational range of the C=O carbonyl group, it is apparent that this band consists of at least two overlapping bands: with a maximum at about 1714 cm^−1^ and with a maximum at about 1732 cm^−1^. It is assumed that the structure of semicrystalline polymers can be described by a three-phase model including mobile amorphous fraction (MAF), rigid amorphous fraction (RAF), and crystalline phase [106,107]. The absorption bands around 1736 cm^−1^, 1720 cm^−1^, and 1714 cm^−1^ have been assigned to C=O in MAF, RAF, and crystalline phase of PBS, respectively [106]. Deconvolution of the carbonyl bands of PBS (Figure 3) as well as composite 4 (Figure 4) demonstrated that they consist of four bands. Assuming that the band at 1736 cm^−1^ originates from the amorphous phase, its percentage of the carbonyl band after PBS ageing increased by 15%, and the percentage of the band originating from the RAF phase increased by 3%. For composite 4, a 3% increase in the C=O band contribution of the MAF phase was calculated, but also a 1.5% increase in the RAF band contribution. For PBS and composite 4, the percentage of C=O bands at about 1718–1712 cm^−1^ (assuming that they originate from the crystalline phase) decreased after ageing by 14% and 6%, respectively. Such calculations may indicate a decrease in the crystallinity of the samples after the ageing test, but they also contradict the crystallinity tests performed with the use of the DSC method. It should be taken into account that in the case of FTIR spectra of the samples after ageing, the carbonyl band may have changed not only due to changes in the crystallinity of the samples but also due to decomposition processes of the ester bond. Such decomposition may have resulted in the formation of carbonyl groups, such as aldehyde groups or carboxyl groups, which may have affected the calculated percentage rates in the C=O band.

#### 3.1.2. Differential Scanning Calorimetry

**Figure 5:**
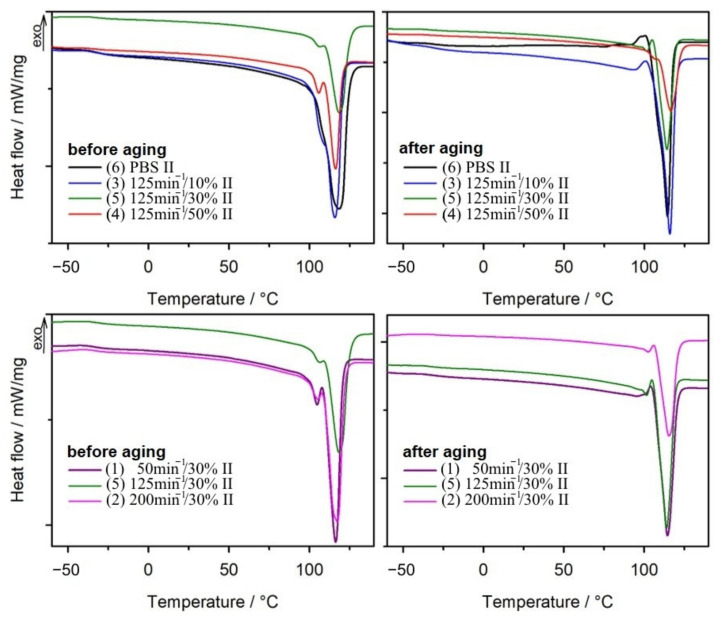
DSC thermograms of the second heating scans for PBS and composites obtained before and after ageing.

**Figure 6:**
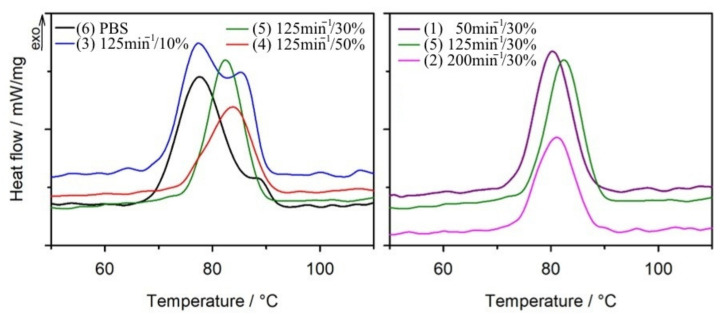
Cooling curves for PBS and its composites obtained after ageing.

All results obtained from the DSC analysis before and after ageing the samples, including glass transition temperature (*T_g_*), crystallization temperature (*T_c_*), melting temperature (*T_m_*), melting enthalpy (Δ*H_m_*), as well as the calculated degree of crystallinity (*X_c_*), are summarized in Table 2. As can be seen from Table 2, it was not possible to observe the glass transition temperature for all tested samples during the first heating scan. The structure of the samples could contain absorbed water and possibly low molecular weight organic compounds formed as a result of the degradation of composites in the ageing test conditions, which is evidenced by the broad endothermic peak on the DSC curves from the first heating scan. Therefore, when considering the effect of composition and conditions of preparation of composites on thermal properties, we relied on the results from the second heating cycle (Figure 5). The *T_g_* values for neat PBS after ageing increased from −32 °C to −27 °C. On the other hand, no significant change in *T_g_* values after ageing was observed for the composites. The change in *T_g_* value for pure PBS is related to the increase in the degree of crystallinity of the polymer after ageing [108]. An increase in crystallinity degree was also observed for the composites, but due to the presence of bran particles, which separate the polymer chains, no change in *T_g_* values was observed. Interestingly, the cooling curves show exothermic peaks originating from crystallization process (Figure 6). In case of PBS and sample 3, which contains only 10% of filler, two maxima on curves of approximately 77 °C and 85 °C are clearly visible, while before the ageing process, only one crystallization temperature (approximately 86 °C) was observed for these materials (Table 2) [109]. This is probably due to the existence of two types of crystals in the structure of the samples. On the basis of FTIR spectra, it can be expected that changes in the chemical structure of PBS took place during ageing, consisting in breaking of polymer chains within ester bonds. This led to the formation of fractions of the polymer with shorter chains, which have a greater capacity for mobility and thus increase their ability to form an ordered phase. As a result, this contributes to lower crystallization temperatures [103]. Moreover, after the ageing process, a clear increase in the degree of crystallinity is observed not only for PBS and composite 3, but also for all other composites. FTIR analysis of the chemical structure of the composites after ageing showed that the polymer chain disintegration reaction occurred in every tested sample. This resulted in the formation of PBS chains with smaller molar masses, which are more easily organized into a crystalline phase. On the other hand, amorphous regions of polymers are more susceptible to degradation than crystalline ones [104,110,111]. The decomposition of the chains present in the amorphous phase caused their release from the amorphous region and they could then arrange themselves into crystalline structures. This ultimately led to an increase in the degree of crystallinity of the samples after ageing, this increase reaching up to 48% for the composite containing 10% bran. With more bran, the increase in the degree of crystallinity was not as great due to limitations in chain mobility caused by the large presence of filler.

The *X_c_* values clearly increased after the ageing process, while no significant changes were observed in *T_m_*. Only for neat PBS is a decrease in *T_m_* evident compared to *T_m_* before ageing. In addition, the endothermic peak in the DSC curve originating from the melting of the crystalline phase after ageing is narrower than for PBS before ageing (Figure 5), without a broadened arm toward lower temperatures. This is due to the increased crystallinity of the sample. DSC curves from second heating scan for all tested materials after ageing show another difference in comparison with curves before ageing (Figure 5). A small endothermic peak preceding the actual melting peak was observed in the melting temperature region before ageing, which we attributed to the melting of less perfect PBS crystallites [75]. Previously reported studies on the melting process of semicrystalline PBS also showed a similar melting behaviour [112,113,114,115]. This complex behaviour is explained by melting recrystallization mechanism, whereby more defective crystals melt at a lower temperature, then further heating of the sample leads to their recrystallization, fusion and further melting at a higher temperature. After the composite ageing process, the first melting peak decreased significantly, which can be attributed to the increase in the degree of crystallinity of the materials.

Considering the changes in the parameters given in Table 2 before and after ageing for materials 1, 2, and 5 (obtained at different screw speeds and the same bran content), changes in the degree of crystallinity of the samples are evident. In each case, the degree increased; the increase of *X_c_* was consistent with the decreasing value of *n*. It is clear that the breakdown of polymer chains into shorter units is responsible for the increase of *X_c_*. In this case, it can be assumed that the homogeneity of bran distribution in the PBS network also affects the ageing process of the composites. Composite 2, obtained at the highest screw speed, shows the most homogeneous distribution of the biofiller in the PBS matrix among samples 1, 5, and 2 [75]. In its case, the increase of *X_c_* after ageing is only 12%, while sample 1, obtained with the smallest *n*, showed an increase of *X_c_* by 45%. The shorter polymer chains of PBS, formed by ageing of the samples, have a greater ability to rearrange into crystalline structures, and the bran acts as a nucleating agent. It can be claimed that heating the samples above the melting point and cooling again led to an increase in the homogeneity of the composite structure, and shorter PBS chains formed crystallites in the vicinity of the bran particles.

#### 3.1.3. Transverse Shrinkage

**Figure 7:**
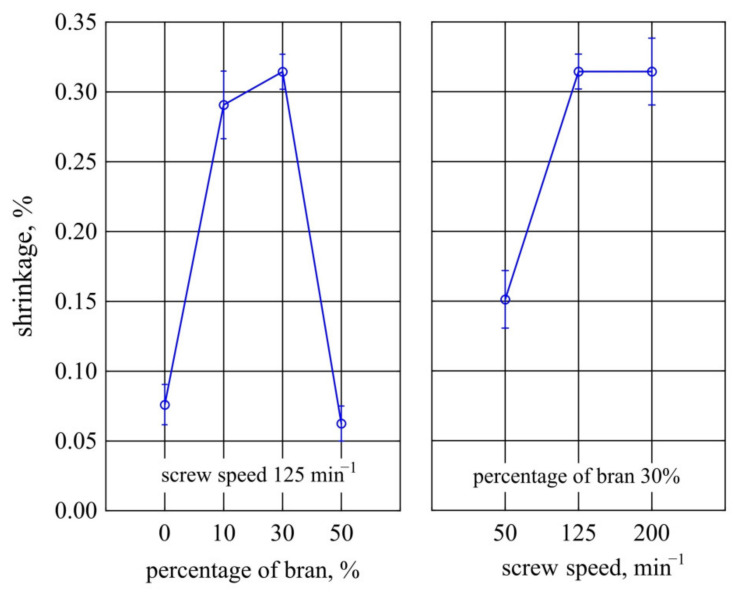
Transverse shrinkage of mouldings due to ageing as a function of bran mass content and extruder screw speed.

The effects of the bran content by weight in the polymeric composition and the screw speed during its manufacturing on the transverse shrinkage of the moulded parts as a result of ageing are shown in Figure 7. The values of transverse shrinkage of mouldings made of PBS and polymer compositions, resulting from ageing, were relatively small and ranged from 0.05% to 0.35%. Post-hoc tests of the obtained results showed that as a result of ageing the transverse shrinkage of mouldings with 10% and 30% bran content is comparable and also significantly higher than that of samples made of PBS alone and of compositions with 50% bran content, which also do not differ significantly from each other. The effect of screw speed on shrinkage proved significant when increased from 50 min^−1^ to 125 min^−1^. When the screw speed was further increased to 200 min^−1^, the shrinkage remained unchanged.

The observed changes in the linear dimensions of the samples are directly related to the effect of PBS degradation due to ageing and the increase in the degree of crystallinity. During injection moulding, the samples undergo processing shrinkage, which is caused partly by thermal expansion effects, but mainly by the crystallization process. In a previous work [75], *p-v-T* studies showed that the crystalline phase of PBS has a significantly lower specific volume than its amorphous phase, which significantly affects the processing shrinkage values [109]. Arrangement of polymer chains into crystalline structures is a long-term process and although the largest increase in shrinkage is observed during cooling immediately after processing, small changes in linear dimensions can still be observed for several months [116]. The decrease in molecular weight of PBS due to ageing facilitates the rearrangement of macromolecules by increasing the degree of crystallinity, and the greater the degree of crystallinity, the greater the shrinkage of PBS [117]. Moreover, it was also shown that the filler particles act as a promoter of crystallization. This means that the arrangement of the crystalline phase occurs around the filler particles and the resulting volume loss exerts compressive stresses on them, which would further affect the shrinkage value [118,119]. At lower filler contents, the crystalline regions around the WB particles will be larger, whereas at 50% content, there will be more crystallization nuclei, but with smaller sizes, as the presence of filler will also limit the mobility of macromolecules [120,121].

#### 3.1.4. Thermal Resistance

**Figure 8:**
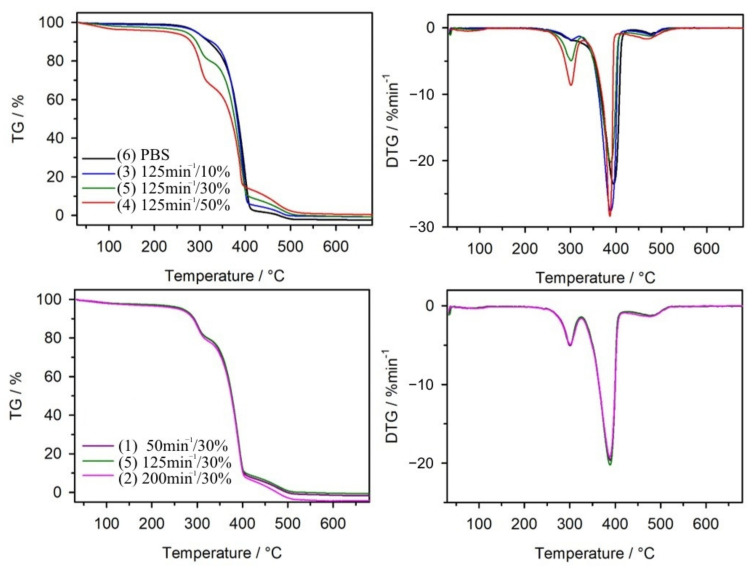
TG and DTG curves for PBS and its composites obtained after ageing.

The thermal resistance of PBS and its composites after artificial ageing was tested in an oxidizing atmosphere. Figure 8 shows TG and DTG curves collected into two groups, depending on the amount of bran in the composite and depending on the extrusion process conditions. Table 3 shows the parameters characterizing the thermal resistance of the discussed materials examined before and after the ageing test. From the data presented, it is evident that artificial ageing altered the thermal resistance of PBS and composites. The course of TG and DTG curves obtained after ageing for composite samples is similar to the course of these curves before ageing [111]. The DTG curves still show three distinct peaks with maxima around 300 °C, 390 °C, and 480 °C. They suggest that the thermal decomposition of the composites proceeded in three stages. However, the *T_max_*_1_ and *T_max_*_2_ values are slightly shifted towards lower temperatures compared to the pre-ageing values. The situation is a little different for PBS. Before ageing, it underwent thermal decomposition in a two-stage process, while after ageing, an additional peak at about 300 °C appears on the DTG curve. This coincides with the first peak in the DTG curves for composites and corresponds to a mass loss of almost 9%. Therefore, it can be concluded that the structural fragments formed during the ageing of PBS are decomposed at about 300 °C; these are most likely short chains of PBS formed by photochemical degradation, terminated by carboxyl groups. The temperature taken as the onset of sample weight loss, *T*_5%_, decreased after ageing for all composites and PBS. The smallest decrease was observed for PBS, while the largest for composite 4 containing 50% bran. It is likely that artificial rain during the ageing process led to partial hydrolysis of the carbohydrate components of the bran. Similarly, the *T*_50%_ values after ageing also decreased slightly compared to the values before ageing. We have previously demonstrated that the composites experienced bran degradation in the first stage of thermal decomposition (at *T_max_*_1_) [76,110]. After ageing, given the weight loss for pure PBS, one might expect that this step is also related to the breakdown of the polymer, not just the bran.

**Figure 9:**
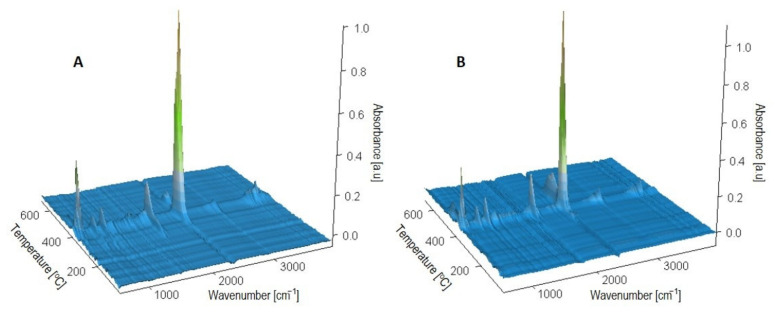
3D-FTIR diagrams of gaseous degradation products of (**A**) PBS and (**B**) composite 4 (containing 50% *w*/*w* of bran).

**Figure 10:**
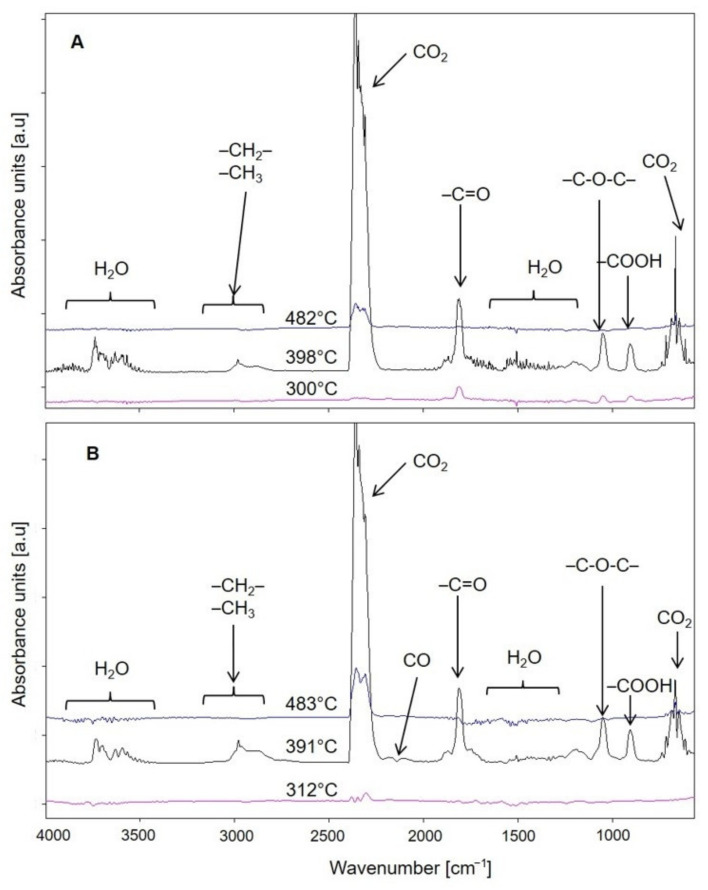
Extracted in maxima of emission FTIR spectra of gaseous degradation products of (**A**) PBS and (**B**) composite 4 (containing 50% *w*/*w* of bran).

FTIR analysis of the gaseous degradation products of the samples allows us to infer the possible mechanism of their thermal degradation. Figure 9 shows 3D FTIR diagrams of the gas decomposition products of PBS and composite 4, while Figure 10 shows FTIR spectra at emission maxima. On the spectrum collected at the first stage of PBS decomposition at 300 °C, low intensity absorption bands are visible at 1812 cm^−1^, originating from vibrations of C=O group, at 1055 cm^−1^ (vibrations of –C–O–C– groups) and at 907 cm^−1^ (vibrations of carboxyl group) as well as bands in the range 2980–2880 cm^−1^ characteristic for vibrations of methyl and methylene groups. These are absorption bands characteristic of succinic acid and butane-1,4-diol in the gas phase [122,123]. In addition, carbon dioxide (absorption bands at 2359–2310 cm^−1^ and 669 cm^−1^) and water (broad bands at approximately 4000–3500 cm^−1^ and 1800–1300 cm^−1^) were observed. These bands suggest that thermal decomposition of PBS after ageing starts with hydrolysis of ester bonds leading to release of succinic acid and butane-1,4-diol and is accompanied by oxidation process evidenced by the release of CO_2_ and water. On the spectrum collected at 312 °C for composite 5, mainly absorption bands from carbon dioxide, carbon monoxide (2180 and 2114 cm^−1^) and water are present. In the vibration region of the carbonyl group around 1812 cm^−1^, a band of negligible intensity can be found. The FTIR spectra for all composites look similar. Thus, oxidation processes are the main ones that take place in their case. However, at the second stage of decomposition, at a temperature about 390 °C, absorption bands characteristic for succinic acid and butane-1,4-diol are observed on the spectra of the composites, indicating, besides the dominant oxidation process, also polyester hydrolysis. The spectrum of PBS in the second decomposition stage is similar to that of composites. On the other hand, only absorption bands from water and carbon dioxide resulting from oxidation processes are present in the spectra of both PBS and composites in the last stage of decomposition, at temperatures around 480 °C.

#### 3.1.5. Colour

**Figure 11:**
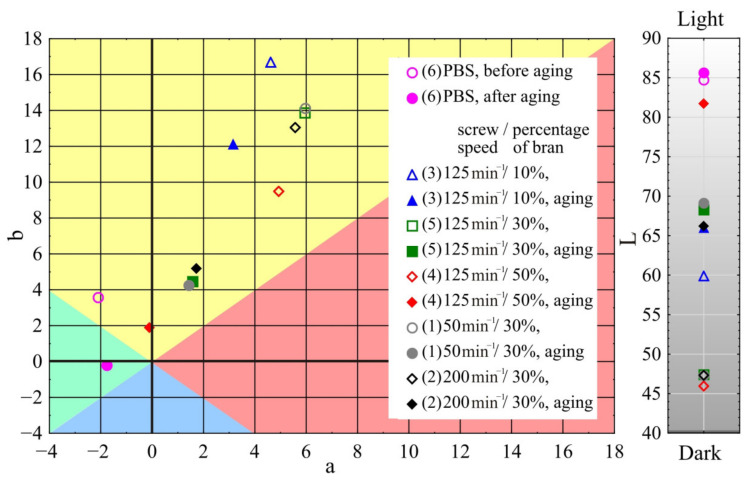
Variation of L, a, b colour parameters before and after ageing of mouldings made of polymer compositions depending on the bran mass fraction content and processing screw speed.

The results of the measurement of L, a, b colour parameters before and after ageing of the mouldings made of the polymer compositions under study are shown in Figure 11. For comparative purposes, the Figure 11 presents also the results obtained for mouldings made of PBS alone without the addition of bran. As a result of ageing, the smallest colour change among the studied samples was observed in mouldings made of PBS alone. The calculated value of ΔE was 3.9, which allows us to define the colour change as distinct (3.5 < ΔE < 5). As a result of ageing, the colour of PBS mouldings changed toward blue shade—mainly decrease in b parameter, with negligible changes in a and L parameters. The effect of ageing on the change of the colour of mouldings produced from compositions containing bran was clearly greater. At bran content as low as *u* = 10%, the colour change amounted to ΔE = 7.8, which allows us to classify it as high (ΔE > 5). For samples of compositions with higher bran contents *u* = 30% and 50%, the colour change was even greater and amounted to ΔE = 23.3 and 36.9, respectively. It has been observed that as a result of the ageing process the colour of the mouldings made of compositions containing bran changes toward blue and green shades (parameter b and a decrease, respectively) and simultaneously the mouldings become lighter/faded, which is evidenced by an increase in the luminance value L. These changes intensify with increasing bran content in the composition. However, no significant influence of the extruder screw speed during the preparation of the composition pellets on the colour of the mouldings produced from it as well as on the colour difference between them after ageing was found.

Comparison of the mouldings before ageing showed that with increasing bran content their colour changes toward blue shades and darkens, the b parameter decreases and the luminance L decreases. These differences are large and in the case of increasing bran content from 10% to 30%, they are ΔE = 12.8 and ΔE = 15.7 when bran content is increased from 10% to 50%.

**Figure 12:**
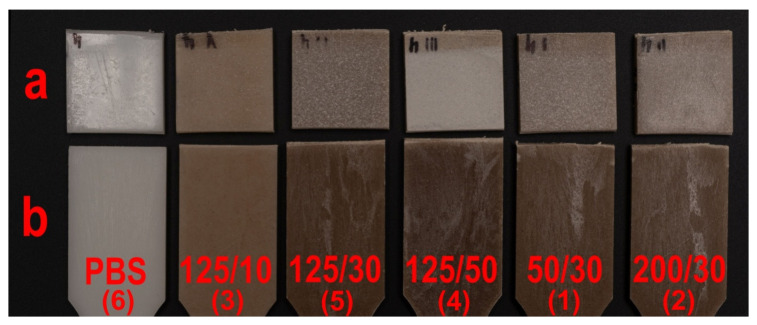
Surface appearance of tested samples after ageing (**a**) and before ageing (**b**).

The obtained colour changes for PBS samples containing wheat bran are directly related to the degradation of the main WB building blocks, namely lignin and cellulose. The key to the course of their degradation is the simultaneous use of UV irradiation and water spraying in the chamber. Lignin itself shows hydrophobic nature and is quite resistant to degradation in water and solutions of weak concentrations [86,124]. However, it shows susceptibility to photodegradation by radiation activity, especially in the presence of water and at elevated temperatures. Depolymerisation of lignin exposes cellulose, which exhibits a hydrophilic nature and significantly increases surface wettability. The presence of water causes swelling of filler grains enabling deeper penetration of radiation and simultaneously accelerates oxidation reaction being the effect of photodegradation [125,126]. In addition, the vast majority of chromophores in lignocellulosic materials is located in lignin macromolecules; therefore, its decomposition will show a significant effect on colour loss [127]. Moreover, cyclic water spraying in the ageing chamber causes mechanical leaching of lignocellulose degradation products and exposure of PBS surfaces or deeper WB fragments [126]. Degradation of filler grains and their removal from the surface of samples as a result of ageing causes a shift in the colour parameters of biocomposites towards the colour of neat PBS. The above analysis is confirmed by the surface appearance of the samples before and after ageing. Figure 12 clearly shows that the fading is focal, and the number of focal spots increases with increasing filler content, covering virtually the entire surface of the composite containing 50% WB.

#### 3.1.6. Surface Roughness

**Figure 13:**
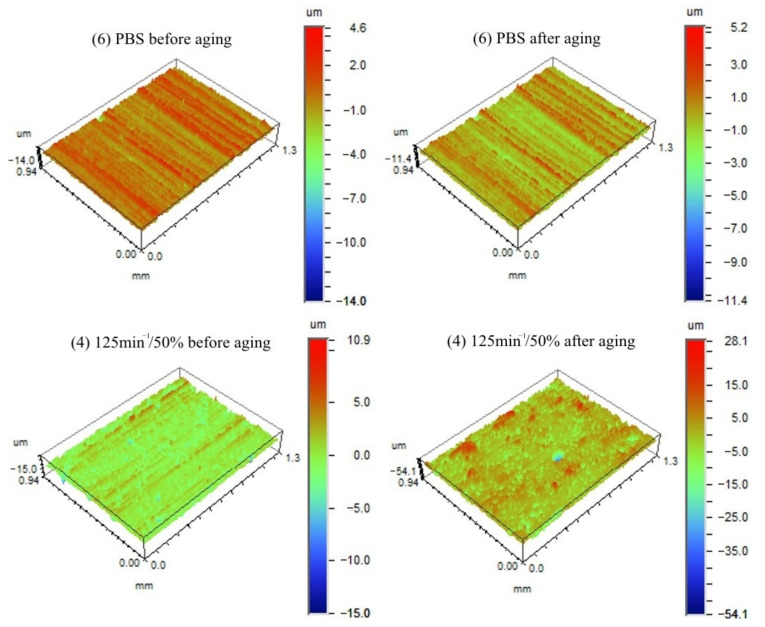
3D images of the surface topography of the PBS and the composite containing 50% bran before and after the ageing test.

**Figure 14:**
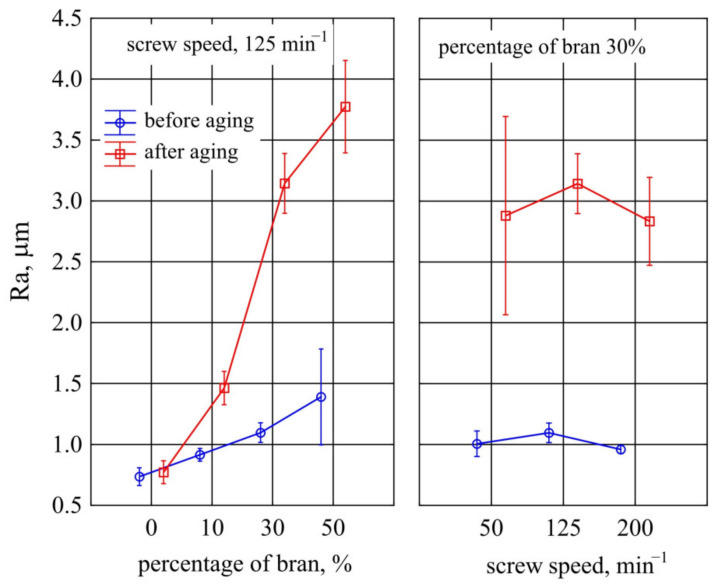
Graph of changes in Ra parameter for PBS and composite containing 50% bran (4) before and after ageing test.

Optical profilometer tests were employed to observe morphological changes in the samples before and after the accelerated ageing test. The 3D images of the surface topography of the PBS and the composite containing 50% bran before and after the ageing test are shown in Figure 13. In order to quantify the roughness changes of the materials, the *R_a_* parameter was calculated, and the results are shown in Figure 14. Neat PBS has the lowest *R_a_* value, both before and after ageing. As the amount of bran in the samples increases, the *R_a_* value also increases. The bran particles, which are visible as blue dots in Figure 13, are responsible for the increase in surface roughness of the composites. Considering the composites containing 30% bran obtained at different values of *n*, no significant effect of this parameter on *R_a_* value was found. A very good homogenization of the composite components occurred at that speed, thus reducing *R_a_*. After the ageing test, the surface roughness of the composites increased significantly, and the trend of changes for samples containing different amounts of bran and obtained at different *n* is similar to that before ageing. Interestingly, as the amount of bran increased, the increase in *R_a_* was steeper and the sample surfaces more heterogeneous, as indicated by the large confidence intervals. The situation was different for neat PBS. After the ageing test, the topography of its surface changed slightly.

The unfilled PBS showed the lowest roughness and its surface structure in the form of longitudinal parallel lines shown in Figure 13 constitutes a mapping of the injection mould surface structure obtained during machining. Biocomposites containing WB showed significantly higher roughness. As the filler content increases, the amount of filler particles localized directly on the sample surface statistically increases, which also increases the roughness. This is a typical result for PBS matrix composites with powder filler [128,129]. The obtained post-ageing roughness results are consistent with the above analysis of the colour results, and the obtained course of changes is directly related to the degradation of the lignocellulosic filler and the mechanical leaching of the products of this degradation [126].

#### 3.1.7. Gloss

**Figure 15:**
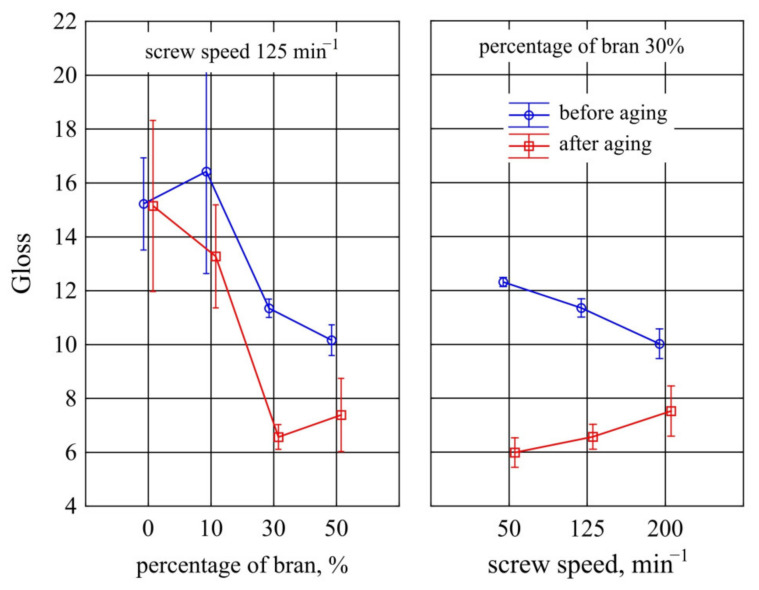
Variation of gloss value before and after ageing of injection mouldings made of polymer compositions depending on the bran mass fraction content and processing screw speed.

Figure 15 presents the results of gloss measurements of mouldings made of the polymer compositions under study and of PBS alone. Statistical analyses did not show any significant influence of the bran content and extruder screw speed during the preparation of the composite granules on the gloss of the mouldings before ageing. A post-hoc test indicated only a difference in gloss between the lowest value of 50 min^−1^ and the highest value of 200 min^−1^ (at constant content of 30%). One of the reasons for such outcome is a large scatter of obtained results especially visible for PBS alone and for compositions with 10% bran content. A statistically significant effect of ageing on the gloss of mouldings was observed for the 30% bran content. In the case of the lower content of 10% bran and PBS without its addition, the gloss before and after ageing did not differ significantly.

The gloss value indicates the ability and mechanism of a surface to reflect incident light in a specular direction. Whether a surface reflects, scatters, or absorbs light depends on the surface roughness, but also on the heterogeneity of the material and surface. The presence of the filler, and then also its composition, size, and quality of distribution in the matrix influence the increase of randomness of light reflection directions and decrease of gloss values [130,131]. Therefore, the introduction of bran powder, as expected, resulted in a decrease in gloss value. On the other hand, the decrease in gloss as a result of ageing is a consequence of the degradation of filler grains located on the surface of the samples, which increases the surface roughness. Consequently, no difference was observed in the gloss of unfilled PBS before and after ageing.

#### 3.1.8. Weight Loss

**Figure 16:**
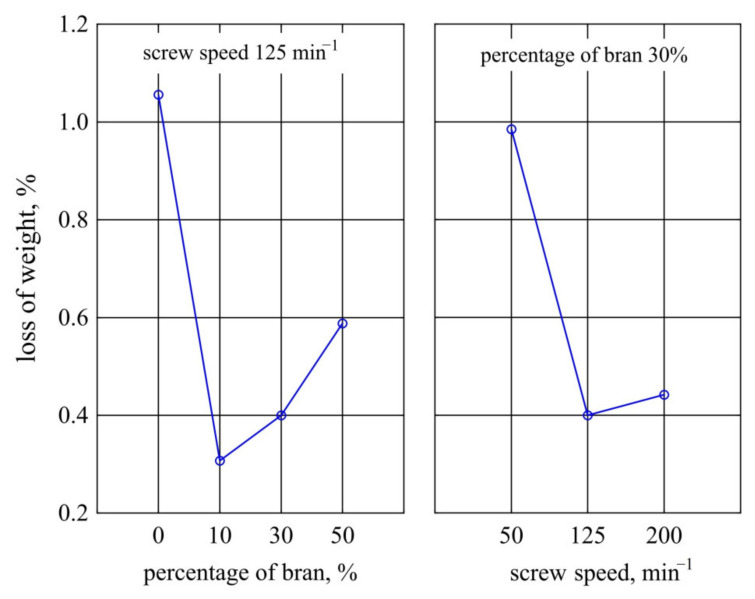
Weight loss resulting from ageing of injection mouldings made of polymer compositions depending on the bran mass fraction content and processing screw speed.

The largest weight loss after ageing was observed for samples made from PBS alone and was 1.06% (Figure 16). Introduction of 10% bran into the composition resulted in a significant decrease in weight loss to 0.31%. Increasing their content caused a successive increase in the value of weight loss to a maximum of 0.59% at 50% bran content. At the same time, a significant decrease in weight loss of the samples prepared from the composition obtained at processing screw speeds higher than 50 min^−1^ was found.

The obtained dependence of weight loss after ageing on bran content remains fully consistent with previous observations. As the bran content increases, the amount of WB particles located on the surface statistically increases, with the particles degraded and washed away during cyclic water spraying [126]. A surprising observation was that the largest weight loss due to ageing was recorded for unfilled PBS, although a weight loss of 1% due to accelerated ageing in the chamber for neat PBS was also obtained by other authors [62]. A potential reason could be that WB particles provided a physical barrier against radiation [125,126]. Due to the numerous chromophores in its macromolecules, lignin is responsible for absorbing about 80–95% of the light incident on lignocellulosic materials, thus it concentrates degradation on the filler present on the sample surface [132,133]. While in unfilled PBS, the entire surface is uniformly exposed to degrading agents. The profilometer tests did not show any difference in the PBS roughness before and after ageing, but some reduction in the height difference between the extreme points can be seen in Figure 13, which may suggest a uniform degradation across the surface. The effect of screw speed, on the other hand, may be related to intensive shearing and fragmentation of filler grains occurring at high speeds, which makes their distribution more homogeneous and the grains smaller [75]. Significantly higher uncertainty of roughness measurements for composites produced at *n* = 50 min^−1^ may suggest a larger dispersion of filler grain sizes.

#### 3.1.9. Hardness

**Figure 17:**
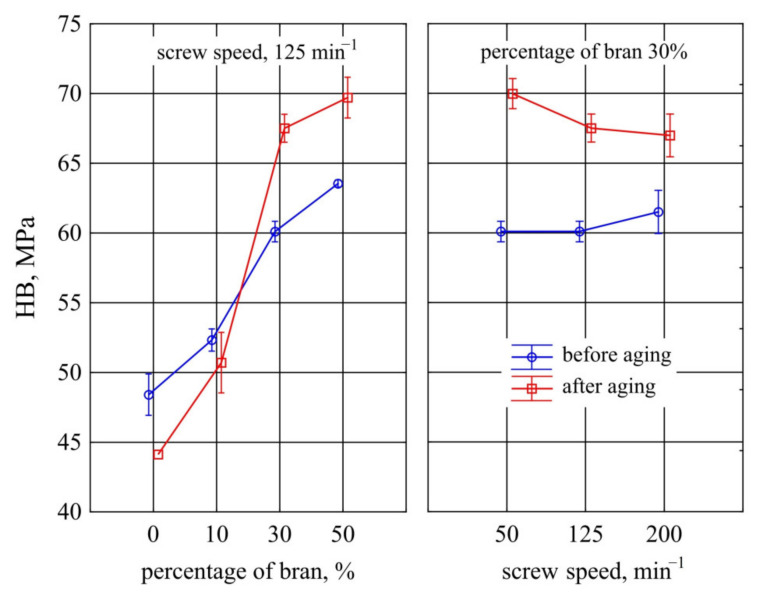
Variation of hardness *HB* through ball indentation method before and after ageing of injection mouldings made of polymer compositions depending on the bran mass fraction content and processing screw speed.

It was observed that the mass content of bran *u* introduced into the composite had a significant effect on the hardness of the mouldings, measured using ball indentation method (Figure 17). Even before the ageing process, 10% of their content caused significant increase of hardness by 3.9 MPa (8%) in comparison to samples with PBS without their addition. Increasing the bran content to 30% resulted in a further 24% increase in hardness compared to PBS. Further increase in bran content to 50% did not cause any significant changes in hardness. The statistical analyses carried out did not show any significant effect of the processing screw speed during the composite production on its hardness measured using the ball pressing method both before and after the ageing process.

Ageing caused a significant decrease in hardness of samples made from PBS alone by an average of 4.3 MPa (9%). In the case of 10% bran content, no significant differences were found in the hardness of the samples before and after ageing. At higher bran contents of 30% and 50%, a significant increase in age hardness by 7.4 MPa (12%), and 6.2 MPa (10%), respectively, was observed.

**Figure 18:**
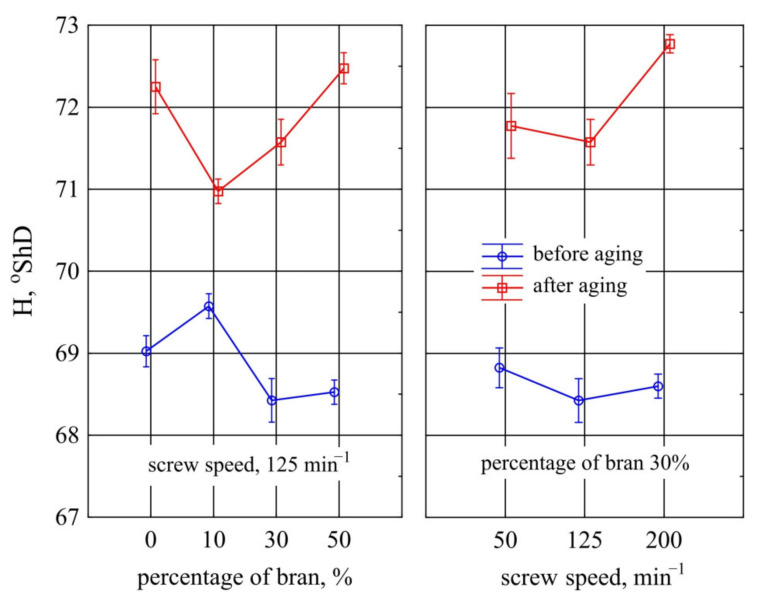
Variation of hardness *H* through Shore D method before and after ageing of injection mouldings made of polymer compositions depending on the bran mass fraction content and processing screw speed.

The tested samples were also subjected to Shore D hardness tests and the results obtained are shown in Figure 18. The hardness results obtained before ageing showed no statistically significant differences for samples with PBS alone and with 10% bran content. Only 30% and 50% bran content caused a very slight but statistically significant decrease in hardness by an average of 0.55 ^o^ShD (0.8%). As well, in the case of this hardness test method, no significant effect of the speed of processing screws during the manufacture of the composition on its hardness before ageing was observed and after ageing a very small increase in hardness occurred only at the highest screw speed.

Ageing caused a significant increase in Shore hardness of all the samples tested. The bran content had a significant effect on hardness after ageing for only 10% of its content—a slight decrease of 1.3 ^o^ShD (1.8%) compared to samples with PBS alone and 1.5 ^o^ShD (2.1%) compared to samples with 50% bran content. The other samples were not significantly different from one another.

The different effect of bran content on pre-ageing hardness for each of the measurement methods used is due to their different characteristics. The ball indentation method involves deflecting a substantial area of the specimen surface with an indenter. The presence of the dispersed phase in the polymer matrix increases the stiffness and restricts the mobility of macromolecules making it difficult to deform [76,77,120]. In the Shore D method, the exerted load is concentrated over a small area, so that transferring the load to filler grains with a hardness less than that of the polymer matrix does not produce a pronounced strengthening effect. Hence, the decrease in hardness in the Shore method at high filler content.

The increase in hardness after ageing is directly related to the increase in degree of crystallinity shown by DSC studies. Due to the close arrangement of macromolecules, the crystalline phase is characterized by higher density, stiffness, and consequently hardness [75,109]. In the case of the ball indentation method, the strengthening effect after ageing is only visible from 30% bran content on. This is due to the penetration of the ball into the outer layer of PBS, which has been degraded by the sum effect of temperature, moisture, and radiation in the ageing chamber. At higher WB contents, the filler particles degrade and begin to cover a significant part of the surface, whose hardness is negligible anyway. In the Shore method, the indenter penetrates the sample to a much greater depth, where the material may not have degraded significantly, so that a strengthening effect due to an increase in crystallinity was observed for all samples differing in content.

### 3.2. Absorption of Polar and Non-Polar Fluids

#### 3.2.1. Water Absorption

**Figure 19:**
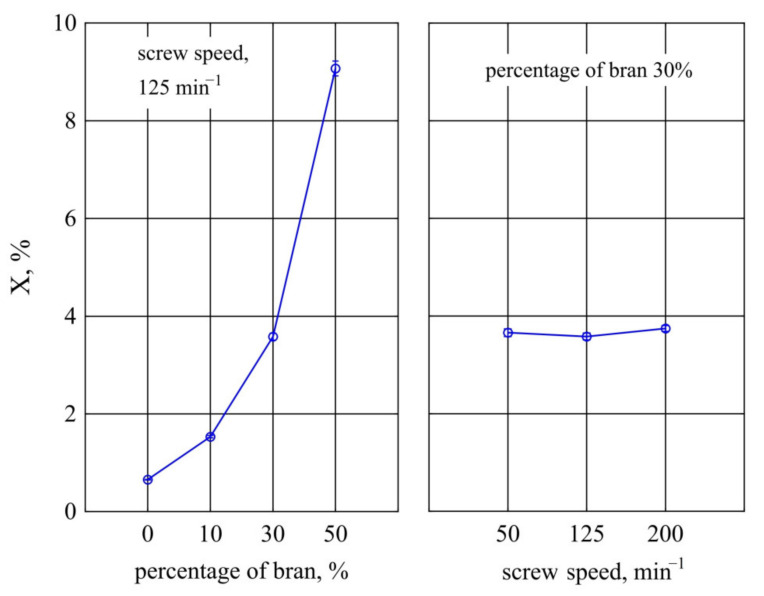
Dependence of water absorption *X* of injection mouldings made of polymer compositions on bran mass content and processing screw rotational speed.

The results of the tests of water absorption *X* from the studied variable factors are presented in Figure 19. The mass content of bran *u* introduced in the compositions studied was found to have a significant effect on the water absorption *X* of the mouldings. Increasing the bran content causes a very large increase in water absorption, which is due to the hydrophilic properties of bran. According to literature data, water retention cap by wheat bran is at the level of 4–4.8 g H_2_O/g, and it may be even higher at a high degree of fragmentation [134,135,136]. Additionally, with an increase in WB content, the distance between adjacent filler particles decreases and the amount of gases, mainly water vapour, emitted during the processing increases, causing the porosity of the composition. This creates opportunities for water to penetrate deeper into the sample structure. The greatest changes, similarly as in the case of the other examined properties, were observed between the compositions which differed most in the bran content of 10% and 50% (DOE layouts 3 and 4). Then, as a result of increasing the bran content, water absorption *X* increased by 7.3% (i.e., to 475% of the initial value). Measurements of samples made from PBS alone showed that their water absorption averaged only *X* = 0.65 ± 0.01%; thus, the bran is primarily responsible for water absorption. The results of statistical analyses showed no significant effect of screw speed on water absorption.

**Figure 20:**
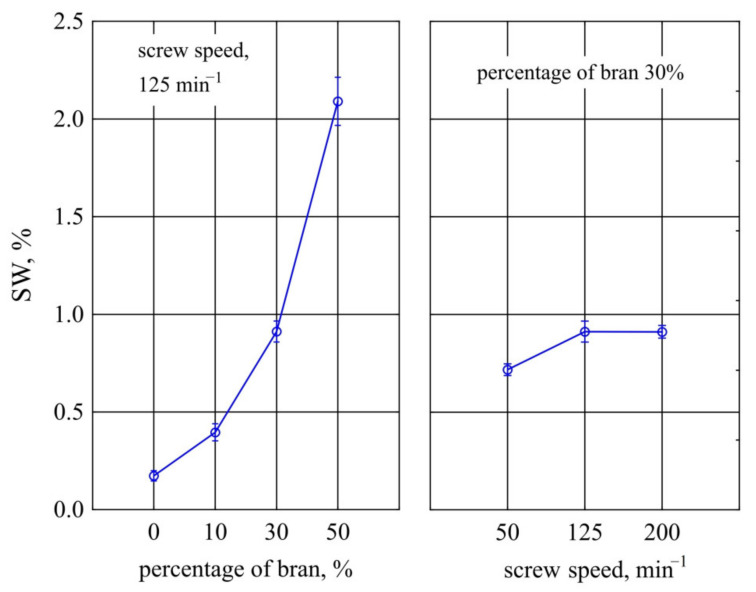
Dependence of swelling *SW* of injection mouldings made of polymer compositions on bran mass content and processing screw rotational speed.

A parameter directly related to water absorption is the change in linear dimensions of a sample due to absorption of a certain volume of fluid. The relationship describing the swelling variation *SW* depending on the bran content in the composition and processing screw speed during its production is shown in Figure 20. The mass content of bran *u* introduced into composition has the greatest influence on this quantity. In this case, the influence of the extruder screw speed *n* during the production of the polymeric compositions under study was clearly smaller, but also statistically significant. Increasing the bran content in the composition causes a significant increase in *SW*, which, similarly to water absorption *X*, is due to hydrophilic properties of bran. The previously mentioned ability of bran to retain about 4 g of water per g of bran must lead to a significant change in volume. The highest increase in *SW* during the tests, i.e., 1.6% (407% of the initial value), was obtained by increasing wheat bran content *u* in the composition from 10% to 50% (DOE layouts 3 and 4). In contrast, the maximum investigated increase in *SW* due to increased screw speed *n* was only 0.2% (i.e., 27% of the initial value). The probable reason for the increase in swelling with screw speed *n* is the fragmentation of soft bran grains due to the action of intense shear stress arising during extrusion of the composition [75]. The conducted analyses did not show the presence of interactions between the studied variable factors. Tests performed on samples made from PBS alone showed that their swelling averaged only *SW* = 0.15 ± 0.04%.

#### 3.2.2. Oil Absorption

**Figure 21:**
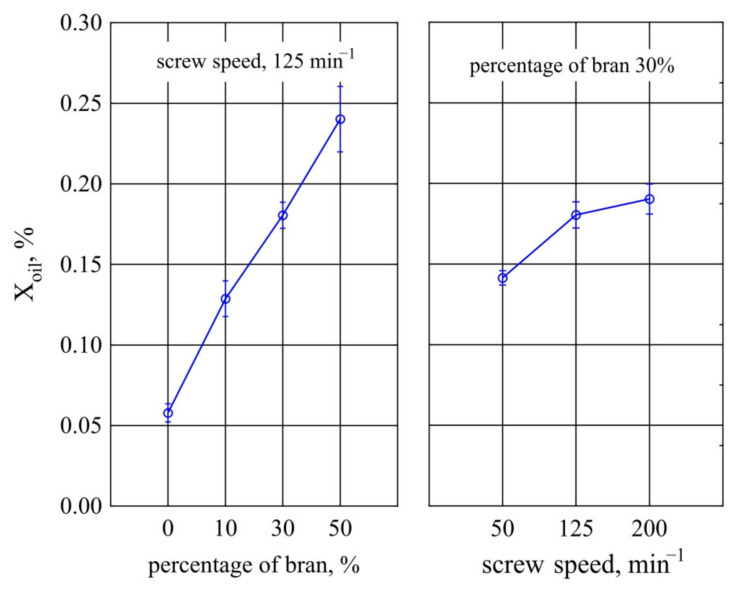
Dependence of oil absorption *X_oil_* of injection mouldings made of polymer compositions on bran mass content and processing screw rotational speed.

Results the tests of oil absorption *X_oil_* of injection mouldings made of the polymer compositions under study and of PBS alone are presented on Figure 21. It has been observed that the highest influence on the oil absorption of the obtained mouldings is exerted by the mass content of bran *u* introduced into the composition, but also by the extruder screw speed *n* during its production. The influence of the two mentioned variable factors is linear. The highest increase in oil absorption *X_oil_* during the tests, i.e., 0.11% (87% of the initial value), was obtained by increasing wheat bran content *u* in the composition from 10% to 50% (DOE layouts 3 and 4). In contrast, the maximum investigated increase in *X_oil_* due to increased screw speed *n* was 0.05% (i.e., 35% of the initial value). Comparative measurements of samples made from PBS alone showed that their oil absorption averages only *X_oil_* = 0.06 ± 0.01%. This means that PBS is a material resistant to vegetable oils, which is also confirmed by the studies of other authors [137]. Therefore, it should be assumed that bran introduced into the composition is mainly responsible for oil absorption, and in particular, the hydrophobic lignin contained in the filler structure [124].

Due to the low oil absorption values, the resulting swelling also took on very low values. The highest increase in linear dimensions was observed for composite 4, containing 50% bran, which was 0.25 ± 0.03%.

### 3.3. Chemical Resistance

#### 3.3.1. Resistance to Acids

**Figure 22:**
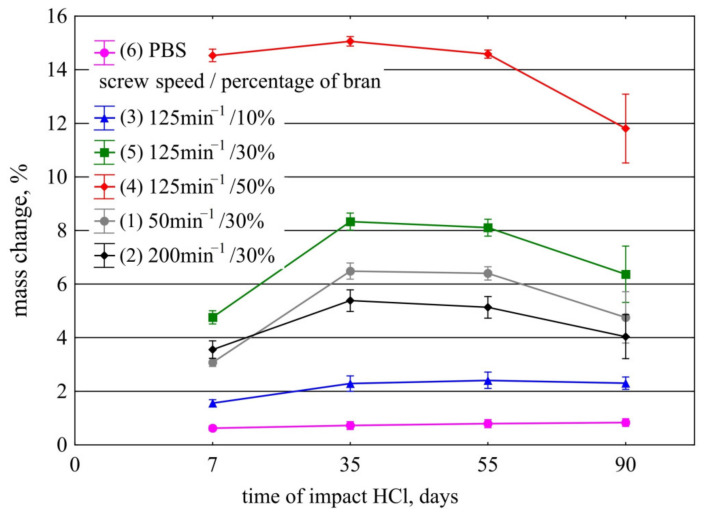
Variation of mass change with hydrolytic degradation time for neat PBS and its composites.

Figure 22 shows the weight changes of the samples during the acidic environment test in 1 M HCl aqueous solution. The observed increase in weight of the samples may seem surprising. It results from the adopted method of measurement. Usually, the mass of samples in chemical resistance tests is measured after they have been dried to a constant mass in an oven. This approach did not work for our study. Drying of the composite samples, after a one-week stay in acid solution, at 55 °C for 48 h, was not sufficient to achieve constant mass. On the other hand, after 48 h of drying, it was clearly visible that the bran was thermally decomposed. Therefore, decision was made for the samples to be dried in a paper towel before weighing them after being removed from the HCl solution. The mass increases observed in Figure 22 are related to the absorption of the acid solution by the PBS and composites. The highest weight gain is observed for the composite containing 50% bran, it decreases according to the decreasing amount of bran in the composites and reaches the lowest value for neat PBS. This relationship is consistent with the water absorption results. It results from the susceptibility of bran to swelling and additionally from the porosity of the samples. In the case of composites, an increase in weight is recorded up to day 35 of the test, except for the sample containing only 10% bran, which displays shows an increase in weight for as long as up to day 55. After 55 days of the samples staying in the acidic solution (after 90 days for sample 3), a progressive weight loss was noted. After 90 days of testing, the weight loss was the highest for the composite with 50% bran content, and the lowest—with 10% bran content. The change in weight of neat PBS over the 90 days of the test remained very similar (approximately 0.8%). Therefore, it can be concluded that the addition of bran to PBS affects the acceleration of chemical degradation in acidic solution. The decrease in weight of the composites is mainly related to the chemical degradation of the bran by acid hydrolysis. Admittedly, PBS belongs to the polyester group and is susceptible to hydrolysis of the ester bond even in pure water. This reaction, however, requires an elevated temperature (approximately 75 °C) [138,139]. Our experiment was conducted at room temperature and even the presence of a strong acid was not sufficient for hydrolysis of neat PBS to occur. The addition of bran susceptible to hydrolysis caused its decomposition, so that the acid solution could diffuse more easily into the internal structure of the composites and react with the PBS chains. Considering the influence of parameter *n* on the resistance of the composites 1, 2, and 5 in an acidic environment, it is apparent that it is not as pronounced as the percentage of bran content in the composites. Composite 5, obtained at a screw speed of 125 min^−1^, showed the greatest weight loss between 35 and 90 days, by almost 2%, while for the other two speeds, the weight loss in this time interval was about 1.5%.

#### 3.3.2. Resistance to Bases

**Figure 23:**
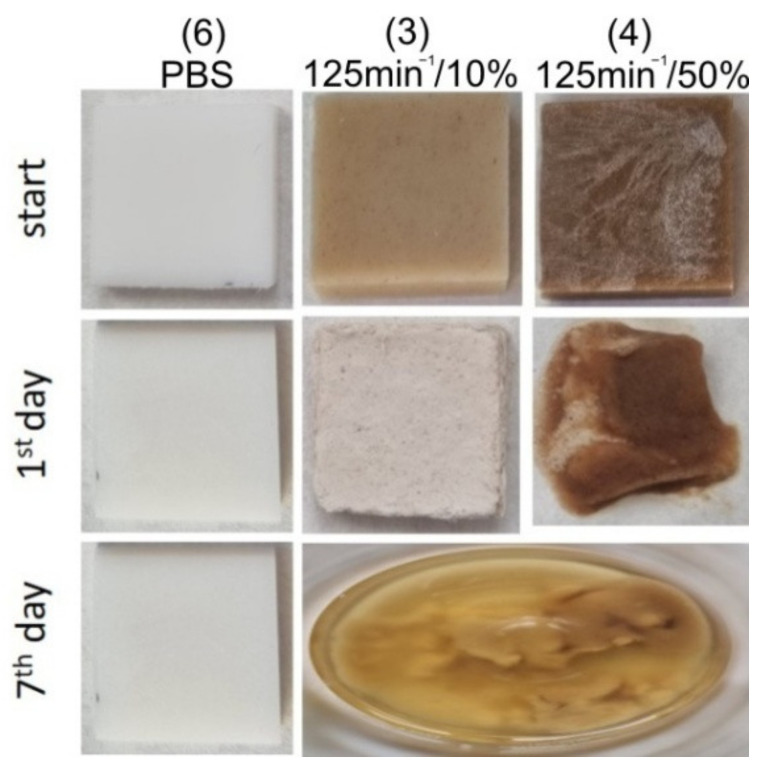
Images of the samples before and during degradation in base solution.

Resistance test of the composites to basic environment in 1 M NaOH aqueous solution showed that the tested samples are not resistant to strong base solution. The composite samples containing 30% and 50% bran disintegrated visibly as soon as after the first day of the test, while the sample with 10% biofiller changed colour and structure, and after another day it also started to disintegrate visibly (Figure 23). After seven days, all composites had completely disintegrated in the base solution. Because the samples became sticky and began to disintegrate early in the test, it was impossible to weigh them. Only the neat PBS sample was integral for 7 days, after which it showed a 3.5% weight loss. The ester bonds in PBS are sensitive to base hydrolysis even at room temperature [140,141]. Bran composed of polysaccharides in the environment of strong bases is easily hydrolysed. As in the case of the action of the acidic solution, their chemical disintegration, enabled faster diffusion of the basic solution into the internal structure of the composite and accelerated the hydrolysis of PBS. In the case of degradation in base solution, only the effect of bran amount on the stability of the composites could be observed while, due to the fast degradation progress, it was impossible to notice the effect of the extruder screw speed.

### 3.4. Biodegradation in Compost

**Figure 24:**
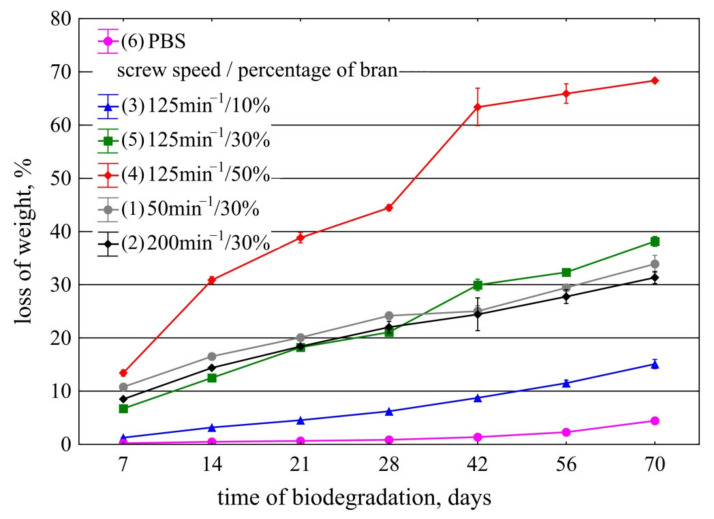
Weight loss resulting from biodegradation of injection mouldings made of polymer compositions depending on the bran mass fraction content and processing screw speed.

The effects of the bran content by weight in the polymeric composition and the screw speed during its manufacturing on the weight loss of the mouldings under controlled biodegradation in compost are shown in Figure 24. The observed changes in the values of mass loss depending on the biodegradation time are linear. The lowest weight loss occurred in samples made with PBS alone and equalled 0.06% per day; after 70 days, the weight of the samples decreased by an average of 4.5%. The weight loss due to biodegradation of samples containing bran was significantly higher and increased with the bran content in the composition. At 10% bran content, it was 0.22% per day, and after 70 days, it averaged 15.1%. At 30% bran content, biodegradation resulted in an average daily weight loss of 0.41% for the three processing screw speeds used during composite production. Changes in weight loss associated with the speed of the processing screws in the production of composites were found to be negligibly small. The greatest weight loss occurred at 50% bran content and averaged 0.9% per day. After 70 days, the average weight loss of these samples was 68.3%.

The degradation kinetics are significantly different for the tested biocomposites and unfilled PBS. Degradation of PBS is relatively slow [62]; therefore, it can be expected that for biocomposites, at least in the initial phase, the degradation mainly involves bran. Due to its chemical structure, WB is more easily enzymatically hydrolysed than PBS and is preferred by microorganisms. As the WB content increases, the rate of weight loss increases significantly with time. This is related to the previously demonstrated high ability of bran to absorb and retain water due to the presence of numerous hydrophilic chemical structures. In addition, the bran grains swelling due to water absorption facilitate the penetration of microorganisms accelerating the degradation of WB. Thus, after the biological decomposition of the filler, the surface of active interaction of water and microorganisms on PBS macromolecules increases [4,142,143,144]. The significant increase in biodegradation kinetics of PBS in compost with increasing lignocellulosic filler content is a typical result also obtained by other authors [52,94,111,144].

## 4. Conclusions

The results presented unambiguously show that the resistance of PBS-based polymer biocomposites filled with wheat bran to physical, chemical, and biological factors depends mainly on the mass content of the filler. The effect of manufacturing conditions of the biocomposite pellets subsequently used for injection moulding of the samples showed moderate or no effect on the properties under study. Thus, from the economic point of view, it will be preferable to produce composite pellets with the highest possible efficiency, as the screw speed in the investigated range does not determine the resistance of the injection mouldings made of them.

Accelerated ageing resulted in changes in the chemical structure of PBS within the ester bond, suggesting macromolecular disintegration. DSC tests performed after ageing showed a significant increase in the biocomposites and PBS degree of crystallinity up to 48%.This phenomenon significantly affects other properties of the tested materials, such as an increase of 0.05–0.35% in shrinkage and a growth of about 10% in hardness. Significant colour changes due to lignin photodegradation were also noted, leading to fading of the samples due to chromophore decomposition. The mentioned filler decomposition was in turn the reason for the changes in surface roughness, the *R_a_* parameter increased of about 168% for biocomposition containing 50% of bran, and weight loss of about 0.31–0.59%. Moreover, the thermal stability of tested materials decreased after ageing of 10 °C for neat PBS and of 30 °C for the composition containing 50% bran.

The biocompositions under study were characterized by significant water absorption in comparison with neat PBS. Both *X* and *SW* parameters increased with increasing filler amount reaching for biocomposition containing 50% of bran of about 9% and 2.1%, respectively. The same relationship was observed for vegetable oil absorption. It was on moderate level, reaching almost 0.25%. It can be concluded that surface grains of lignocellulosic filler, which are composed of both polar and non-polar macromolecules, are responsible for the absorption of both polar and non-polar liquids.

Chemical resistance tests showed that PBS is resistant to acidic environment at room temperature, whereas biocompositions exhibited the mass loss of about 1.5–2% between the 35th and 90th day of test. Nevertheless, samples still retained their integrity after 90 days. The biocompositions, in contrast to neat PBS, exhibited negligible resistance to strong bases, as the samples containing the filler completely lost integrity within seven days. The lack of the resistance on the alkaline environment should be taken into account when the possible applications are planned for the compositions of PBS filled with wheat bran.

Biodegradation studies have shown a significant effect of wheat bran content on the kinetics of degradation of the analysed biocompositions. The most rapid degradation is, of course, in the bran itself, which is the preferred habitat of microorganisms. Unfilled PBS degrades relatively slowly under the test conditions; on the other hand, the composition containing 50% of WB reached 68% disintegrability after 70 days of incubation. Such high disintegration indicates that proposed biocompositions are compostable, which is very advantageous from an environmental point of view.

## Figures and Tables

**Table 1:** Experimental design.

Experimental Design Layout	*n*, min^−1^	*u*, %
1	50	30.0
2	200	30.0
3	125	10.0
4	125	50.0
5	125	30.0
6 PBS	-	0

**Table 2:** DSC data for PBS and its composites obtained before (denoted as b) and after ageing (denoted as a).

Sample	Heating I				Cooling	Heating II			
	*T_g_* (°C)	*T_m_* (°C)	Δ*H_m_* (J/g)	*X_c_* (%)	*T_c_* (°C)	*T_g_* (°C)	*T_m_* (°C)	Δ*H_m_* (J/g)	*X_c_* (%)
PBS b PBS a	−25.6 −25.7	121.3 116.3	72.1 94.9	65.4 86.0	86.4 77.6/88.9	−31.7 −27.6	118.5 113.5	66.7 84.4	60.5 76.5
1(50/30) b 1(50/30) a	−31.3 -	118.9 118.5	51.1 71.9	66.2 93.1	82.2 80.3	−32.1 −32.0	116.3 115.3	38.2 55.6	49.5 72.0
2(200/30) b 2(200/30) a	−31.5 -	118.9 117.7	51.7 49.5	67.0 64.1	81.3 81.8	−32.4 −32.2	117.2 116.3	39.4 44.3	51.0 57.4
3(125/10) b 3(125/10) a	−33.3 −26.6	118.8 118.8	63.3 98.2	63.8 98.9	86.4 77.4/85.3	−33.4 −33.5	115.8 115.0	55.3 81.9	55.7 82.5
4(125/50) b 4(125/50) a	−32.5 −15.3	117.6 118.8	39.0 48.1	70.7 87.2	83.3 83.8	−32.5 −32.4	116.6 116.6	30.6 35.3	54.5 64.0
5(125/30) b 5(125/30) a	−31.4 -	118.0 117.7	41.9 65.5	54.3 84.8	79.6 82.5	−32.3 −31.5	118.2 114.7	38.8 49.8	50.3 64.5

**Table 3:** Parameters characterizing the thermal resistance of PBS and its biocomposites before (denoted as b) and after ageing (denoted as a), obtained based on thermogravimetry (TG) and derivative thermogravimetry (DTG) curves.

Sample	*T*_5%_ (°C)	*T*_50%_ (°C)	*T_max_*_1_ (°C)	Δ*m*_1_ (%)	*T_max_*_2_ (°C)	Δ*m*_2_ (%)	*T_max_*_3_ (°C)	Δ*m*_3_ (%)	*R_m_* (%)
bran	201	303	296	68.0	-	-	459	29.7	2.3
PBS b PBS a	307 297	386 384	- 300	- 8.9	395 394	97.9 88.6	463 477	2.0 2.4	0.1 0.1
1(50 30) b 1(50 30) a	274 260	380 376	303 300	20.5 20.5	392 389	70.3 71.1	476 476	9.1 8.2	0.1 0.2
2(200 30) b 2(200 30) a	276 259	379 375	303 301	20.1 21.8	390 388	70.0 71.1	476 476	9.7 7.1	0.2 0.0
3(125 10) b 3(125 10) a	299 288	383 382	303 301	8.5 9.5	391 388	87.0 85.1	475 478	4.5 5.3	0.3 0.1
4(125 50) b 4(125 50) a	261 231	372 369	300 302	31.5 33.5	386 387	53.5 52.0	462 469	14.7 14.2	0.3 0.3
5(125 30) b 5(125 30)	274 260	381 377	303 300	19.8 20.5	391 388	71.1 70.4	476 476	9.0 9.1	0.1 0

## Data Availability

The data presented in this study are available on request from the corresponding author.

## Abbreviations

PBS	Poly(butylene succinate)
WB	Wheat bran
UV	Ultraviolet
*LCF*	Lignocellulosic filler
*u*	Wheat bran content
*n*	Extruder screw rotational speed
FTIR	Fourier transform infrared (spectroscopy)
DSC	Differential scanning calorimetry
*X_c_*	Degree of crystallinity
Δ*H_m_*	Melting enthalpy
*T_m_*	Melting point
*T_c_*	Crystallization temperature
*T_g_*	Glass transition temperature
TG	Thermogravimetry
DTG	Derivative thermogravimetry
*T*_5%_, *T*_50%_	Temperature of 5% and 50% of mass loss
*T_max_*	Temperature of the maximum rate of mass loss
Δ*m*	Mass loss corresponding to *T_max_*
*R_m_*	Residual mass
*L**	Luminance/brightness
*a**	Colour from green to red
*b**	Colour from blue to yellow
Δ*E*	Difference between the two colors
*R_a_*	Average roughness parameter
*E_jc_*	Specific energy consumption
*p-v-T*	Relationship between pressure *p*, specific volume *v* and temperature *T*
*HB*	Ball indentation hardness
*H*	Shore D hardness
*X*	Water absorption
*SW*	Swelling
*X_Oil_*	Oil absorption
